# Effect of short-term glycemic control and physical activity on health-related quality of life among type 2 diabetes receiving care in a tertiary health facility in Ogun State, Nigeria: a cross-sectional study

**DOI:** 10.11604/pamj.2023.44.47.35680

**Published:** 2023-01-24

**Authors:** Olufemi Oyeleye Oyewole, Olatunde Odusan, Ayotunde Oladunni Ale, Kolawole John Sodeinde

**Affiliations:** 1 Department of Physiotherapy, Olabisi Onabanjo University Teaching Hospital, Sagamu, Nigeria,; 2 College of Health Sciences, University of KwaZulu-Natal, Private Bag X54001, Durban, South Africa,; 3 Department of Medicine, Olabisi Onabanjo University Teaching Hospital, Sagamu, Nigeria,; 4 Department of Community Medicine, Babcock University, Ilishan-Remo, Nigeria

**Keywords:** Glycemic control, health-related quality of life, physical activity, type 2 diabetes

## Abstract

**Introduction:**

there are myriad of factors that influence health-related quality of life (HRQoL) which relationships remain unclear. Some of the factors include glycemic control and physical activity. This study determined the relationship between glycemic control, physical activity, and HRQoL among people living with type 2 diabetes.

**Methods:**

data from a cross-section of persons living with type 2 diabetes included information about their most recent fasting blood glucose (FBG), physical activity (PA), and HRQoL. The PA and HRQoL were assessed with long-form international physical activity and short-form-36 questionnaires, respectively while FBG was gleaned from patients´ records. Data were subjected to statistical analysis at p<0.05 regarded as significant.

**Results:**

a total of 119 participated in the study with mean age of 61.8±11.8 years and mostly women, 60.5% (n=72). About 68.9% (n=82) were physically active, 84.0% (n=100) had poor short-term glycemic control (median blood glucose 134, IQR (108-187) mm/dl). There was a positive correlation between participants´ PA and physical health (r= 0.425, p=0.001), mental health (r= 0.334, p= 0.001) and overall HRQoL (r= 0.403, p= 0.001) but not with FBG (r= 0.044, p= 0.641). However, their FBG correlated with the mental health domain of the HRQoL (r= -0.213, p= 0.021). The physically active had better overall HRQoL (62.53±19.10 vs 50.28±23.10, p = 0.001) than the physically inactive which effect persisted when stratified for glucose control (68.16±19.19 vs 47.62±21.52, p = 0.001). There was however no influence of glycemic control on the relationship between PA and HRQoL [b = 0.000, 95% CI (0.000, 0.000), t = 0.153, P = 0.88] meaning that the relationship is not moderated by glycemic control.

**Conclusion:**

physical activity is beneficial for improved HRQoL in type 2 diabetes irrespective of glycemic control. This calls for increasing the level of awareness and education of type 2 diabetics aimed at improving their physical activity levels and their quality of life.

## Introduction

Diabetes mellitus is a public health issue with increasing prevalence [1-3]. There is a global target to halt the increase in diabetes by 2025 with strategies gear towards access to regular screening, advocacy for regular physical activities, intake of healthy diet, and tobacco avoidance [3]. Lifestyle modifications including Physical Activity (PA) and healthy diet have been recommended for effective management of type 2 diabetes. PA has glycemic moderating properties demonstrated by reduction in glucose levels of People Living with Diabetes (PLWD) [4]. For PLWD, the American Diabetes Association has recommended moderate (with 50-70% increase in maximal heart rate) or moderate to vigorous aerobic exercises for a minimum of 150 minutes spread out over not less than three days a week with a maximum of two consecutive days between exercise sessions [5].

The effect of diabetes on patients has been measured using conventional methods such as biochemical measurements coupled with assessment of morbidity and mortality patterns. A paradigm shift has of late focused on Health-Related Quality of Life (HRQoL) as evaluating the effect of the disease from the patient´s viewpoint [6]. For self-assessment of the impact of management of non-communicable diseases on health, HRQoL is now one of the most evaluated treatment outcomes [7]. PLWD face substantial impairment in their HRQoL as against non-diabetics [8]. This may be partly psychological because PLWD are anxious about their glycemic control vis-a-vis complication. Furthermore, their management and lifestyle modifications that include dietary change and physical exercises influence HRQoL [9,10].

Although PA has been shown to improve HRQoL, glycemic control is important to enhance this effect [11]. Far-reaching scientific proof has revealed how proper and sustained pharmacological management improves the immediate and long-term HRQoL of PLWD [12]. A recent systematic review and meta-analysis assessing the relationship between the level of exercise and glycosylated hemoglobin (HbA1c) (a measure of long-term glucose control) revealed that HbA1c decreases with exercise [13]. However, these changes in HbA1c were associated with the overall duration but not the intensity (METs), time (min/session), or frequency (sessions/week) of exercise [13]. Therefore, it is imperative to establish the effect of short-term glycemic control and PA on HRQoL from patients´ outcome perspectives. With paucity of studies on the effect of PA and glycemic control on HRQoL, this study sought to assess the relationship between glycemic control, PA, and HRQoL among people living with type 2 diabetes.

## Methods

**Study design and setting:** this research was a descriptive cross-sectional study. The study was conducted at Dame Adebutu Diabetes Care Centre of the Olabisi Onabanjo University Teaching Hospital (OOUTH), Sagamu, Nigeria. Created on the 1^st^ of January 1986, this 350-bedded hospital provides tertiary health care services and training in Ogun State southwest Nigeria. The diabetic center where this study was conducted is being coordinated by the endocrinology unit of the Internal Medicine Department of the hospital where specialist endocrinologists give expert care to diabetes patients in collaboration with dietitians, physiotherapists and other experts required for a wholesome diabetic care.

**Study population**: the target population were PLWD. Patients who have been diagnosed with type 2 diabetes according to ADA criteria [14], above 30 years of age and clinically stable attending Dame Adebutu Diabetes Care Centre were included in the study. Exclusion criteria include diseased states that can limit mobility. A standard formular was used to determine sample size with 95% confidence level set at 1.96, expected proportion of physically active in the population at 69% and absolute error/precision at 10% [15,16]. Minimum sample of 82 PLWD are needed to power the study. Therefore, a consecutive sampling technique was used to recruit 119 participants out of 183 patients attending the clinic during the study period.

**Data collection**: the health-related quality of life (HRQoL) was assessed with the Short Form-36 Questionnaire (SF-36). SF-36 consists of 36 items assessing eight health dimensions of physical functioning (10 items), role limitations due to physical problems (4 items), bodily pain (2 items), general health (5 items), vitality (4 items), social functioning (2 items), role limitation due to emotional problems (3 items) and mental health (5 items) [17]. The first four dimensions´ scores are summarized into physical composite domain and the last four dimensions´ scores into mental composite domain [17] The score ranges from 0-100 with higher scores indicating better HRQoL. Physical activity was assessed using the long-form of the International Physical Activity Questionnaire (IPAQ). IPAQ assessed activity in the domain of work, transportation, domestic chores, gardening (yard), and leisure time. PA was categorized as physically active or inactive [18]. The most recent fasting blood glucose values of type 2 diabetes participants were gleaned from patients' hospital records and categorized as short-term good glycemic control (≤100mg/dl) or poor control (>100mg/dl) according to International Diabetes Federation criteria [19]. The questionnaires were self-administered for those who are literate in English or Yoruba and through interviews for those who are not.

**Definitions**: HRQoL is defined as “how well a person functions in their life and his or her perceived wellbeing in physical, mental, and social domains of health” [6]. Physical activity is defined as “any bodily movement produced by skeletal muscles that require energy expenditure” [20].

**Ethical consideration**: Health Research Ethics Committee of Olabisi Onabanjo University Teaching Hospital approved the study (approval no: OOUTH/DA.326/981). All the participants were informed about the details of the study and give informed consent. They were told participation is voluntary and could withdraw from the study at any time.

**Statistical analysis**: data were checked for normality and found to be skewed. Data were subjected to descriptive statistical analysis of mean, median, standard deviation, interquartile, frequency, percentile, and percentage. Differences between groups were assessed using Mann-Whitney U test, correlation between the three variables of interest was determined with Spearman´s correlation coefficient while the moderating effect of glycemic control on physical activity and HRQoL was assessed using moderation analysis. Statistical package for social sciences version 25 was used to analyze the data at P < 0.05.

## Results

**General characteristics of the study participants:** there was a total of 119 study participants with mean age of 61.8±11.8 years and mostly women (72, 60.5%). Most were physically active (82, 68.9%), had poor short-term glycemic control [median blood glucose 134, IQR (108 - 187) mm/dl] (100, 84.0%) with only 17 (14.3%) having good short-term glycemic control (median (inter-quartile)) value of 94.0mg/dl (77.5 - 98.0) (Table 1).

**Table 1:** details of measured variables among study participants

Variables	Range	Mean(SD)	Median	Percentiles			Skewness(SE)	Kurtosis(SE)
				25^th^	50^th^	75^th^		
**HRQol**	14.50-99.17	62.86(22.03)	62.48	45.13	62.48	81.98	-0.23(0.22)	-0.95(0.44)
**Physical Health**	10.00-98.75	59.34(23.90)	58.13	40.63	58.13	81.25	-0.09(0.22)	-0.97(0.44)
**Mental Health**	19.00-100.00	66.37(21.98)	69.00	49.25	69.00	85.50	-0.39(0.22)	-0.87(0.44)
**FBG mg/dl**	56.00-463.00	160.25(78.10)	134.00	108.00	134.00	187.00	1.96(0.22)	4.48(0.44)

**Correlation and mean differences between variables**: the PA score correlated significantly with physical and mental health domains (r= 0.425, p=0.001), (r= 0.334, p= 0.001) and overall HRQoL (r= 0.403, p= 0.001) but not with fasting blood sugar (r= 0.044, p= 0.641). However, fasting blood glucose correlated with mental health domain only (r= -0.213, p= 0.021). Relationship between HRQoL, PA, and short-term glycemic control among participants are shown in Table 2. There was significant difference between overall HRQoL of the physically active when compared with those not active (62.53±19.10 vs 50.28±23.10, P = 0.001) but not between participants with and without glycemic control (66.16±23.17 vs 62.20±21.88, P = 0.496). The physically active when compared with the not active had significantly better HRQoL in both mental health and physical domains (71.62±19.30 vs 54.75±23.33, P = 0.001 vs (65.44±21.09 vs 45.81±24.46, P = 0.001) of HRQoL respectively (Table 2). The effect of PA on overall HRQoL of participants persisted when stratified for glycemic control on participants without short-term glycemic control. There was significant difference between those physically active and those not active (68.16±19.19 vs 47.62±21.52, P = 0.001) with no significant difference noted among those that achieved glycemic control (71.71±18.41 vs 59.93±27.50, P =0.501) (Table 2). The same pattern was observed for physical and mental health domains of the HRQoL. When stratified for PA, there were no significant differences in HRQoL scores of participants with and without short-term glycemic control nor either active or not active (Table 2).

**Table 2:** participants’ health-related quality of life stratified by physical activity and short-term glycemic control

Quality of life	Physical activity			Short-term glycemic control		
	Active mean±SD	Inactive mean±SD	p-value	Control mean±SD	Uncontrol mean±SD	p-value
Physical health	65.44±21.09	45.81±24.46	0.001	61.86±25.04	58.73±23.75	0.557
Mental health	71.62±19.30	54.75±23.33	0.001	70.47±23.40	65.67±21.82	0.376
Overall QoL	62.53±19.10	50.28±23.10	0.001	66.16±23.17	62.20±21.88	0.496
	**Control short-term glycemic**			**Uncontrol short-term glycemic**		
	**Active mean±SD**	**Inactive mean±SD**	**p-value**	**Active mean±SD**	**Inactive mean± SD**	**p-value**
Physical health	68.01±19.17	54.93±30.17	0.312	65.04±21.32	43.30±22.61	0.001
Mental health	75.41±21.64	64.92±25.50	0.441	71.27±19.07	51.95±22.35	0.001
Overall QoL	71.71±18.41	59.93±27.50	0.501	68.16±19.19	47.62±21.52	0.001
	**Active**			**Inactive**		
	**Control mean±SD**	**Uncontrol mean± SD**	**p-value**	**Control mean±SD**	**Uncontrol mean±SD**	**p-value**
Physical health	68.01±19.17	65.04±21.32	0.749	54.93±30.17	43.30±22.61	0.283
Mental health	75.41±21.64	71.27±19.07	0.518	64.92±25.50	51.95±22.35	0.266
Overall QoL	71.71±18.41	68.16±19.19	0.664	59.93±27.50	47.62±21.52	0.283

**Moderation association between variables**: PA and glycemic control account for 14.96% variance on overall HRQoL (R2 = 0.1496, F3 = 6.628, P = 0.001) though interaction between PA and glycemic control was not statistically significant [b = 0.000, 95% CI (0.000, 0.000), t = 0.153, P = 0.88]. This indicates that the relationship between PA and HRQoL is not moderated by glycemic control (Table 3). Similar patterns were observed for physical and mental health.

**Table 3:** moderating analysis of short-term glycemic control on effect of physical activity on health-related quality of life

Quality of life	B	CI	T	p-value	R^2^		
						F	p-value
**Physical health**	0.00	0.00 – 0.00	0.35	0.73	0.1569	7.01	0.001
**Mental health**	0.00	0.00 – 0.00	0.68	0.50	0.1273	5.49	0.002
**Overall QoL**	0.00	0.00 – 0.00	0.15	0.88	0.1496	6.63	0.001

## Discussion

This study evaluated the relationship between glycemic control, PA, and HRQoL among people living with type 2 diabetes. There was a positive correlation between participants´ physical activity and physical health, mental health, and overall HRQoL but not with fasting blood glucose. The physically active had better overall HRQoL than the physically inactive which effect persisted when stratified for glucose control. There was however no influence of glycemic control on the relationship between physical activity and HRQoL. The mean age of diabetic patients in the current study was 61.8±11.8 years similar to age group of 45-64 years earlier reported to have the highest prevalence of type 2 diabetes [21] confirming diabetes as a disease of the middle age and elderly and in consonance with the age prevalence of type 2 diabetes mellitus in Nigeria and other parts of the world [22-24]. Physical activity among this study participants revealed no correlation with their fasting blood glucose while the overall HRQoL and glycemic control were significantly different among those physically active and those not active. It is important to note that there are other factors that may be responsible for blood glucose control besides physical activity. Marin-Penalver had earlier postulated that people living with diabetes need a long period of adherence to lifestyle changes and anti-diabetic drug use to achieve good glycemic control [25]. According to Murano *et al*. a combination of physical exercise, anti-diabetic drugs, and dietary modifications are the three key strategies to good glycemic control while another study from Korea by Park J-H *et al*. revealed other factors significantly associated with glycemic control to include income level, duration of diabetes, presence of co-morbidities like hypertension coupled with anthropometric measurements like waist circumference [26,27]. Any or all these factors might be responsible for the presence of high prevalence of both physical activity level and poor glycemic control among our study participants. Nonetheless, our study observation of a positive relationship between glycemic control and physical activity is in consonance with other reports [4,5,28].

Quality of life encompasses physical, social, and mental domains. It takes into consideration the individual perspective of overall wellness [29]. While studies have shown that diabetes mellitus impact negatively on the different domains of quality of life, physical activity has a direct relationship with quality of life [30,31]. According to Cassilhas, physical activity is efficient in promoting brain health in both normal and diseased states as it protects against intellectual deterioration [32]. Furthermore, Aschalew *et al*. observed that exercise was positively associated with the physical and psychological domains of HRQoL of patients with diabetes [10]. This study noted direct relationship between physical activity and HRQoL of people living with diabetes as well as improved physical and mental health observed with increased physical activity. Many reasons have been adduced to the positive effect of exercise on HRQoL and include improvement in the effectiveness of the immune system, protection against different diseased conditions, helping to reduce stress, controlling weight, strengthening the heart, improving sleep, and controlling cholesterol levels [33].

There are reports that a significant moderator could affect the strength of the direct effect of the predictor, physical activity, on the variable HRQoL [34]. An assessment of glycemic control as potential moderator that could enhance or weaken the association between physical activity and HRQoL in this study revealed no influence. Our findings however have some clinical implications suggesting that notwithstanding their glycemic control, people living with type 2 diabetes should be encouraged to engage in physical activity for improved HRQoL. Therefore, education about physical activity during clinic attendance will go a long way to stimulating regular participation in physical activity and thereby improve quality of life. The limitations of this study include the study duration and its reliance on fasting blood glucose, a measure of short-term control, rather than HbA1c, a measure of long-term control. The study findings should therefore be interpreted with caution. A longitudinal study using HbA1c with similar studies from other centers is suggested to enhance the generalizability of our findings.

## Conclusion

Our data suggest better HRQoL in various domains among physically active people living with diabetes, irrespective of glycemic controls. This calls for increased awareness and education on improving physical activity among people living with diabetes for improved quality of life.

### What is known about this topic

- *Moderate physical activity reduces glucose level in type 2 diabetes;*
- *People with type 2 diabetes have impaired HRQoL;*
- *Physical activity improves HRQoL in type 2 diabetes*.

### What this study adds

- *Fasting blood glucose correlate negatively with mental health domain of HRQoL;*
- *Fasting blood glucose does not moderate relationship between physical activity and HRQoL;*
- *Irrespective of glycemic control, physical activity is beneficial for improved HRQoL*.
